# tmRNA-mediated trans-translation as the major ribosome rescue system in a bacterial cell

**DOI:** 10.3389/fgene.2014.00066

**Published:** 2014-04-07

**Authors:** Hyouta Himeno, Daisuke Kurita, Akira Muto

**Affiliations:** Department of Biochemistry and Molecular Biology, Faculty of Agriculture and Life Science, Hirosaki UniversityHirosaki, Japan

**Keywords:** tmRNA, SmpB, ribosome, *trans*-translation, molecular mimicry

## Abstract

Transfer messenger RNA (tmRNA; also known as 10Sa RNA or SsrA RNA) is a small RNA molecule that is conserved among bacteria. It has structural and functional similarities to tRNA: it has an upper half of the tRNA-like structure, its 5’ end is processed by RNase P, it has typical tRNA-specific base modifications, it is aminoacylated with alanine, it binds to EF-Tu after aminoacylation and it enters the ribosome with EF-Tu and GTP. However, tmRNA lacks an anticodon, and instead it has a coding sequence for a short peptide called tag-peptide. An elaborate interplay of actions of tmRNA as both tRNA and mRNA with the help of a tmRNA-binding protein, SmpB, facilitates *trans*-translation, which produces a single polypeptide from two mRNA molecules. Initially alanyl-tmRNA in complex with EF-Tu and SmpB enters the vacant A-site of the stalled ribosome like aminoacyl-tRNA but without a codon–anticodon interaction, and subsequently truncated mRNA is replaced with the tag-encoding region of tmRNA. During these processes, not only tmRNA but also SmpB structurally and functionally mimics both tRNA and mRNA. Thus *trans*-translation rescues the stalled ribosome, thereby allowing recycling of the ribosome. Since the tag-peptide serves as a target of AAA^+^ proteases, the *trans*-translation products are preferentially degraded so that they do not accumulate in the cell. Although alternative rescue systems have recently been revealed, *trans*-translation is the only system that universally exists in bacteria. Furthermore, it is unique in that it employs a small RNA and that it prevents accumulation of non-functional proteins from truncated mRNA in the cell. It might play the major role in rescuing the stalled translation in the bacterial cell.

## INTRODUCTION

**FIGURE 1:**
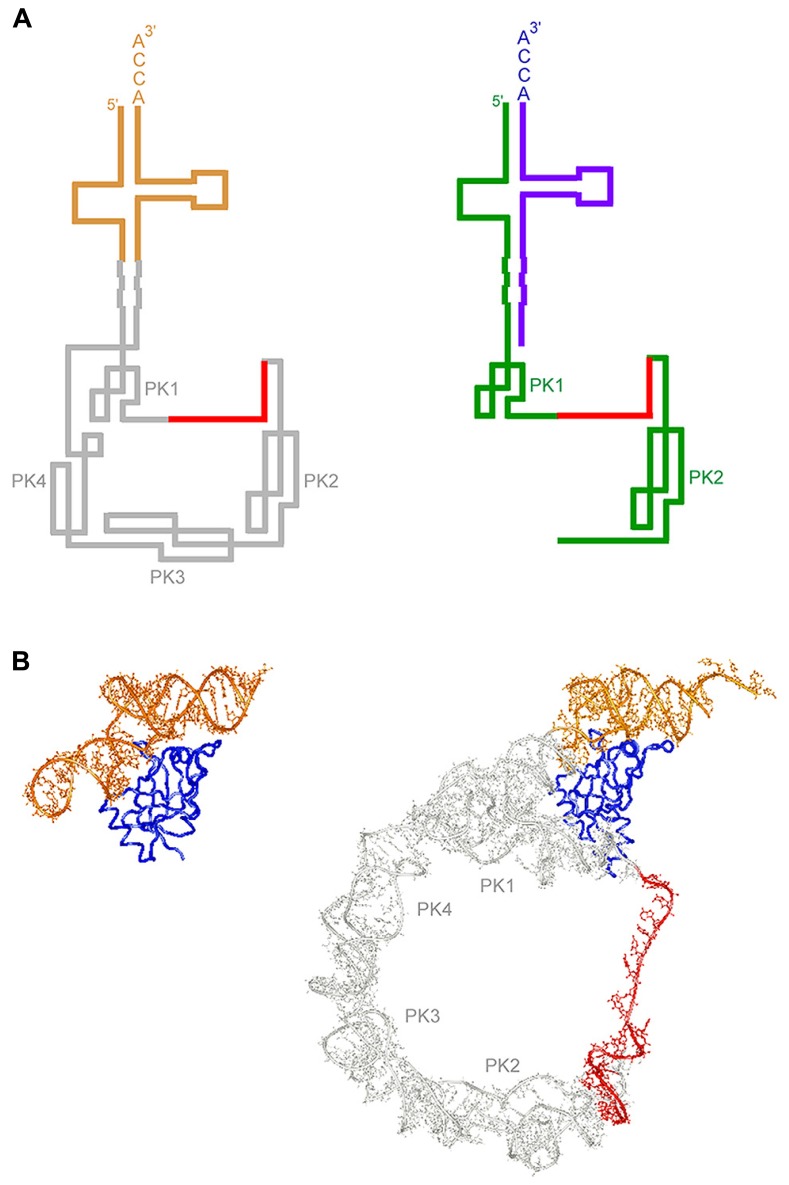
**Secondary and tertiary structures of tmRNA.** **(A)** Secondary structure of tmRNA. (Left) The most typical secondary structure of tmRNA is shown, with TLD and the tag-encoding region designated in brown and red, respectively. (Right) A secondary structure of two-piece tmRNA. The 5’-coding piece and 3’-amino acid acceptor piece are designated in green and violet, respectively. **(B)** Tetiary structure of tmRNA or its fragment in complex with SmpB. (Left) A crystal structure of a complex of a TLD fragment lacking the 3’CCA end of *T. thermophilus* tmRNA (the 5’ 25 residues and 3’ 34 residues connected by a UUCG loop) and the globular domain (N-terminal 123 of a total of 144 residues) of SmpB (PDB ID: 2CZJ; Bessho et al., 2007). TLD (brown) and SmpB (dark blue) mimic the upper and lower halves of the L-shaped structure of tRNA, respectively. (Right) A cryo-EM structure of tmRNA·SmpB in a *T. thermophilus* post-translocated state complex of* trans*-translation (PDB ID: 3YIR; Weis et al., 2010b) is shown, with TLD, the tag-encoding region and SmpB designated in brown, red, and dark blue, respectively. The N-terminal globular domain of SmpB mimicking the lower half of the L-shaped structure of tRNA is in close contact with the upstream region of the tag-encoding sequence.

**FIGURE 2:**
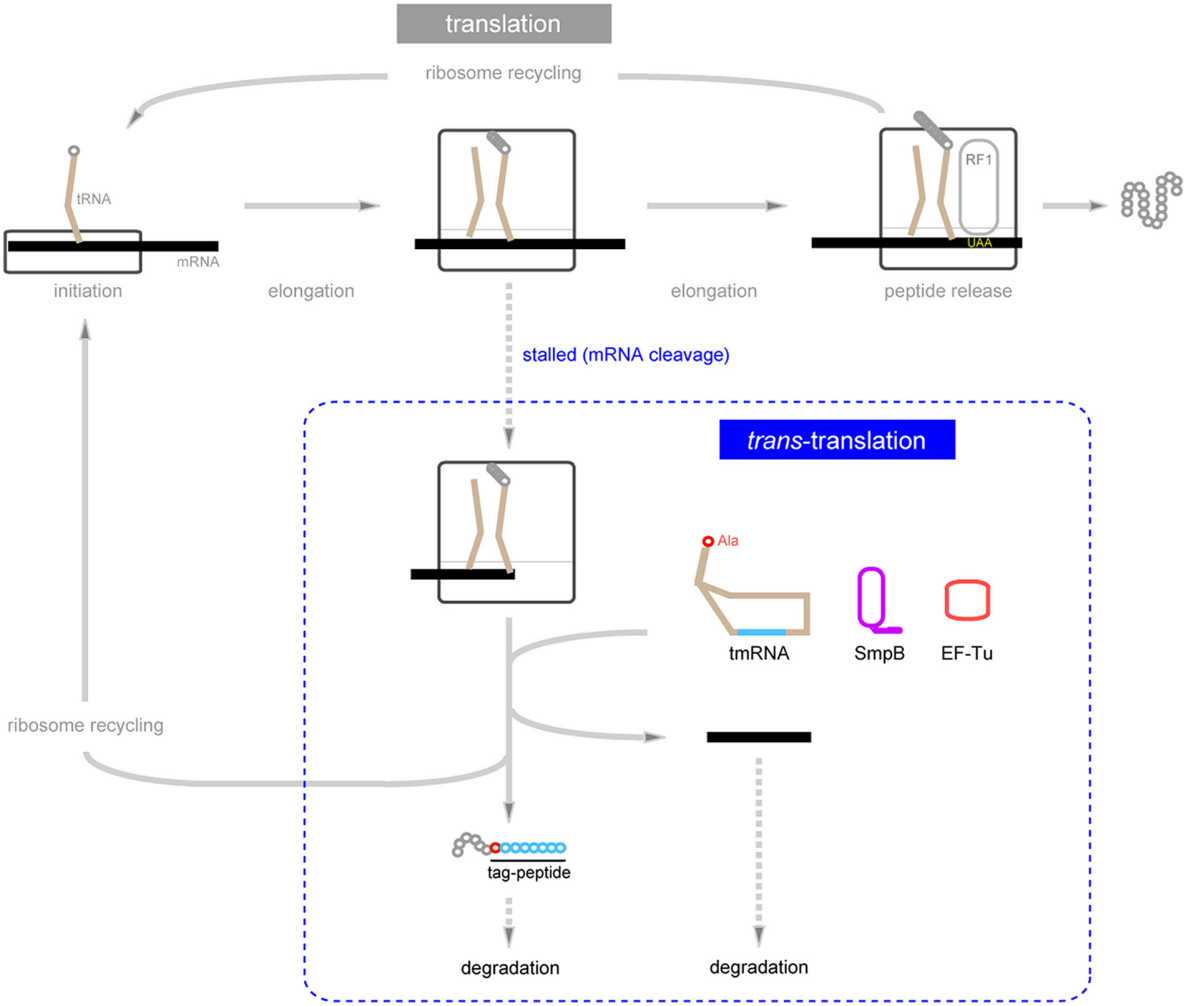
**Schematic representation of *trans*-translation.** When the translating ribosome is stalled due to mRNA truncation, it is rescued by *trans*-translation, which produces a chimeric protein consisting of the C-terminally truncated polypeptide (gray circles) encoded by 3’-truncated mRNA fusing the tmRNA-encoded short peptide (blue circles) with an alanine residue (red circle) originating from alanine aminoacylated to the 3’-end of tmRNA in between them.* Trans*-translation promotes degradation of both truncated mRNAs and aberrant polypeptides from truncated mRNAs.

Translation often stalls in various situations in a cell, sometimes in a programmed fashion and other times unexpectedly. For example, translation of mRNA lacking a stop codon (non-stop mRNA) does not terminate efficiently because peptide release factor does not function. Thus, the cell should have a system to cope with such emergencies. However, little attention was given to this issue until the mid-1990s, and therefore the discovery of tmRNA was a big surprise. Initially the tRNA-like structure and function of tmRNA were elucidated. Both terminal regions of tmRNA can form a secondary structure resembling the upper half of the cloverleaf-like structure of tRNA (Komine et al., 1994; Ushida et al., 1994), which includes several tRNA-specific consensus sequences and base modifications (**Figure 1A**; Felden et al., 1997, 1998). Like that of tRNA, the 3′ end of tmRNA can be aminoacylated with an amino acid (alanine) by an aminoacyl-tRNA synthestase (alanyl-tRNA synthetase; Komine et al., 1994; Ushida et al., 1994). Other tRNA-like functions, such as 5′ processing by RNase P (Komine et al., 1994), binding to EF-Tu (Rudinger-Thirion et al., 1999; Barends et al., 2000, 2001; Hanawa-Suetsugu et al., 2001) and interaction with 70S ribosome (Ushida et al., 1994; Komine et al., 1996; Tadaki et al., 1996), have also been revealed. Although it is about fivefold larger than tRNA, tmRNA has no apparent anticodon, making it difficult to clarify whether and how tmRNA is involved in translation. A few years later, it was found that tmRNA has functions not only as tRNA but also as mRNA: a short peptide is encoded by the middle part of tmRNA (Tu et al., 1995), which is surrounded by four pseudoknot structures (**Figure 1A**; Nameki et al., 1999b,c). Intriguingly, these two functions cooperate, rather than being independent, to produce a chimeric polypeptide from two mRNAs, a C-terminally truncated polypeptide encoded by mRNA fusing the tmRNA-encoded short peptide with an alanine residue of unknown origin in between them (Keiler et al., 1996; Muto et al., 1996; Himeno et al., 1997). This acrobatic translation involving co-translational mRNA swapping produces a single polypeptide from two mRNAs, and thus it has been called *trans*-translation (**Figure 2**; Muto et al., 1998). This system provides a stop codon to allow completion of translation of a non-stop mRNA and consequently recycling of the ribosome. In addition, *trans*-translation has been regarded as a quality control system that prevents non-functional polypeptides derived from truncated mRNAs from accumulating in the cell, as the tag-peptide consisting of the first alanine residue and the tmRNA-encoded short peptide, especially the sequence of the last four hydrophobic amino acids (ALAA), serves as the target for cellular ATP-dependent proteases (Gottesman et al., 1998; Herman et al., 1998; Flynn et al., 2001; Choy et al., 2007).

*Trans*-translation requires a tmRNA binding protein called SmpB in addition to canonical elongation factors (Karzai et al., 1999). SmpB consists of a globular domain and an unstructured C-terminal tail (Dong et al., 2002; Someya et al., 2003). It binds to the tRNA-like domain (TLD) of tmRNA to prevent tmRNA from degradation and enhance aminoacylation of tmRNA (Barends et al., 2001; Hanawa-Suetsugu et al., 2002; Shimizu and Ueda, 2002; Nameki et al., 2005). SmpB also plays a crucial role in the ribosomal process of *trans*-translation.

The *trans*-translation system is universally present in bacterial cells and is present in organelles of some eukaryotes but not in the cytoplasm of eukaryotes or archaebacteria. There is accumulating evidence indicating that the bacterial cell is equipped with additional systems to cope with stalled translation. Here, we review the current understanding of the molecular mechanism and the cellular functions of tmRNA-mediated *trans*-translation as well as other ribosome rescue systems.

### Molecular Mechanism of *Trans*-Translation

**FIGURE 3:**
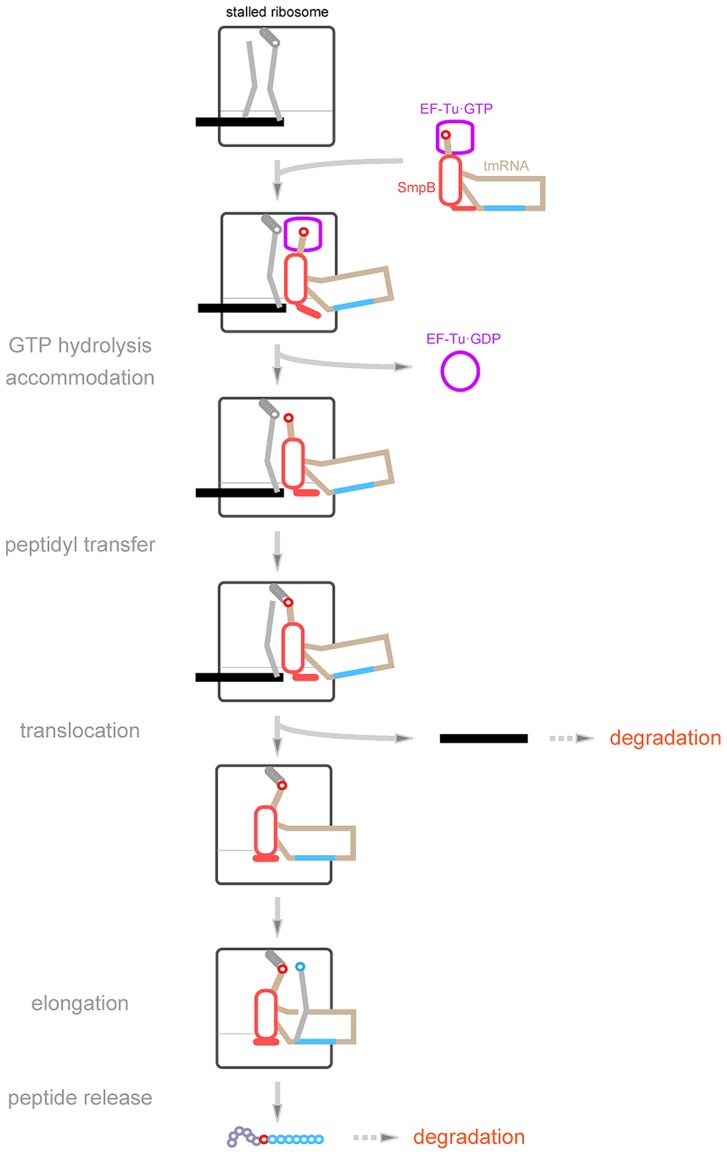
**A current model of *trans*-translation.** Ala-tmRNA·SmpB·EF-Tu·GTP enters the vacant A-site of the stalled ribosome to trigger *trans*-translation. GTP hydrolysis by EF-Tu may induce a conformation change of the stalled complex, allowing the C-terminal tail of SmpB to interact with the mRNA path with an extended structure. After peptidyl-transfer, peptidyl-Ala-tmRNA·SmpB translocates from the A-site to the P-site with the help of EF-G to drive out truncated mRNA from the ribosome. During this process, the extended C-terminal tail somehow folds. Then the resume codon of tmRNA is set on the decoding region. SmpB and the tag-encoding region are shown in red and blue, respectively.

An outline of the process of *trans*-translation is as follows (**Figure 3**): initially, tmRNA in complex with SmpB is aminoacylated with alanine by alanyl-tRNA synthetase. Ala-tmRNA enters the A-site of the stalled ribosome on a truncated mRNA to receive the nascent polypeptide from peptidyl-tRNA in the P-site. Then peptidyl-Ala-tmRNA translocates to the P-site, which exchanges the template from truncated mRNA to the tag-encoding region on tmRNA. It can reasonably explain the missing origin of the alanine residue connecting the truncated polypeptide encoded by mRNA with the tmRNA-encoded tag-peptide: it is derived from the alanine moiety aminoacylated to tmRNA. This model was proposed on the basis of the results of an *in vivo* study showing that truncated polypeptides fusing the tmRNA-encoding tag-peptide in its C-termini accumulate in the cell when a truncated mRNA is expressed (Keiler et al., 1996), and the model was supported by the results of an *in vitro* study showing that the tag-peptide is synthesized using *Escherichia coli* cell extract depending on the addition of poly(U) and on the aminoacylation capacity of tmRNA (Muto et al., 1996; Himeno et al., 1997). However, several questions have been raised. How does tmRNA find the stalled ribosome? How does tmRNA enter the A-site of the ribosome without an anticodon? How does the tag-encoding region of tmRNA substitute for truncated mRNA? How is the resuming point on tmRNA determined? SmpB has emerged as the key molecule to solve these questions.

Besides canonical translation factors and tmRNA, SmpB is the minimal requirement for *in vitro trans-*translation (Shimizu and Ueda, 2002; Takada et al., 2007; Kurita et al., 2012). SmpB has been thought to continue binding to tmRNA throughout the process of *trans*-translation (Ivanova et al., 2007). In a crystal structure of a complex of SmpB and a model RNA fragment corresponding to TLD of tmRNA, the globular domain of SmpB binds to TLD so that it compensates for the lack of the lower half of the L-form structure in tmRNA (**Figure 1B**; Gutmann et al., 2003; Bessho et al., 2007). Thus, TLD in complex with the globular domain of SmpB would structurally mimic a whole tRNA molecule. A directed hydroxyl radical probing study has revealed two SmpB binding sites in an *E. coli *ribosome, one in the A-site and the other in the P-site, both of which can be superimposed on the lower half of the tRNA molecules in the translating ribosome (Kurita et al., 2007). An additional mimicry of the upper half of tRNA by TLD could complete two whole translating tRNA mimics at the A-site and P-site so that their aminoacyl ends are oriented to the peptidyl-transferase center. The pattern of cleavage of 16S rRNA by hydroxyl radicals from the C-terminal tail residues has suggested two binding sites of the C-terminal tail of SmpB with two different modes of conformation in the ribosome in addition to the unstructured conformation in solution: an extended conformation from the A-site to the downstream tunnel along the mRNA path as an α-helical structure and a folded conformation around the mRNA path in the P-site (Kurita et al., 2007, 2010).

On the basis of these SmpB properties, the *trans*-translation process can be described in more detail (**Figure 3**). Ala-tmRNA·SmpB·EF-Tu·GTP enters the vacant A-site of the stalled ribosome. GTP hydrolysis induces a conformational change of the stalled complex to release EF-Tu.GDP, allowing accommodation of Ala-TLD·SmpB in the A-site. During this process, the C-terminal tail of SmpB interacts with the mRNA path extending towards the mRNA entry channel. Subsequently, Ala-TLD in the A-site receives the nascent polypeptide chain from peptidyl-tRNA in the P-site, and the resulting peptidyl-Ala-TLD·SmpB translocates from the A-site to the P-site. During this process, the C-terminal tail of SmpB dissociates from the mRNA entry channel and binds around the site of codon–anticodon interaction in the P-site with change in its conformation from the extended structure to the folded structure, which in turn promotes release of mRNA from the ribosome. The conformational change of the C-terminal tail concomitant with translocation makes the A-site free, thereby allowing introduction of the resume codon of tmRNA into the decoding region. This model has been supported by results of structural studies of several *trans*-translation intermediates. Cryo-EM studies have revealed three kinds of intermediates in the pre-accommodated, accommodated, and translocated states (Kaur et al., 2006; Cheng et al., 2010; Fu et al., 2010; Weis et al., 2010a,b). In all of them, SmpB and TLD occupy the lower and upper halves, respectively, of the tRNA-binding sites. Although the C-terminal tail of SmpB has not been identified in these maps due to low resolution, its interaction with the mRNA path has clearly been shown in a crystal structure of a pre-accommodation state complex of *trans*-translation containing kirromycin (Neubauer et al., 2012).

This model can explain why *trans*-translation preferentially occurs at the ribosome stalled on mRNA with a shorter 3′-extension, which has been exemplified *in vitro* (Ivanova et al., 2004; Asano et al., 2005): the C-terminal tail of SmpB competes with the 3′-extension of mRNA for the mRNA entry channel. A chemical footprinting study has suggested that SmpB interacts with A1492, A1493, and G530 in 16S rRNA, which form the decoding region (Nonin-Lecomte et al., 2009). However, these nucleotides can be changeable without loss of both peptidyl-transferase and GTP hydrolytic activities in *trans*-translation, indicating much lower significance of these decoding nucleotides for *trans*-translation (Miller et al., 2011). In a crystal structure of a *Thermus thermophilus* pre-accommodation state complex of *trans*-translation, G530 stacks with a residue (Y127) around the start of the C-terminal tail of SmpB (Neubauer et al., 2012). Recently, Miller and Buskirk (2014) have found that the corresponding residue in *E. coli* SmpB (H136) has a crucial role in GTP hydrolysis, leading to the proposal that stacking of this residue with G530 triggers GTP hydrolysis.

EF-G promotes release of truncated mRNA from the stalled ribosome after peptidyl transfer to Ala-tmRNA, suggesting the presence of a canonical translocation-like step in the *trans*-translation process (Ivanova et al., 2005). During this event, SmpB must pass through the barrier between the A-site and P-site, and tmRNA must enter the inside of the mRNA entry channel to set the resume codon in the decoding region. Consistently, in a cryo-EM map of a translocational intermediate complex containing EF-G and fusidic acid, both bridge B1a, which serves as a barrier between the A-site and P-site, and latch, which is usually closed by the interaction between the head (helix 34) and body (G530 region) to form the mRNA tunnel, are open (Ramrath et al., 2012). Precise positioning of the resume codon at the decoding region requires the sequence just upstream of the resume codon at positions –6 to +1 (Williams et al., 1999; Lee et al., 2001), and this sequence is recognized by the globular domain of SmpB (Konno et al., 2007), suggesting that SmpB bridges two separate domains of tmRNA in the P-site to determine the resume codon for tag-translation presumably just after translocation. This is in agreement with cryo-EM maps of translocated state complexes of *trans*-translation (**Figure 1B**; Fu et al., 2010; Weis et al., 2010b). Another study has suggested the importance of the C-terminal tail of SmpB and its interaction with the start GCA codon on tmRNA for determination of the start point of tag-peptide translation (Camenares et al., 2013). Taken together, the results suggest that the interaction between tmRNA and SmpB is more important for resume point determination than the interaction between tmRNA and the ribosome. It should be noted that some kinds of aminoglycosides that bind the decoding region shift the resume point of tag-translation (Takahashi et al., 2003; Konno et al., 2004).

Although several examples of the potential molecular mimicry of tRNA by a translation factor have been reported, SmpB is the sole molecule that has been assumed to mimic the dynamic behavior of tRNA throughout all of the classical and hybrid states, A/T, A/A, A/P, P/P, and P/E, in the translating ribosome. The ribosomal protein S1, which has been identified as a tmRNA-binding protein (Wower et al., 2000), is not thought to participate in the early stage of *trans*-translation, at least until the first translocation (Qi et al., 2007; Takada et al., 2007).

### Requirement of mRNA Cleavage for *Trans*-Translation

Because the tag-sequence serves as a degradation signal, *trans*-translation products are hardly detected in the cell or its extract, although they become accumulated and thus detectable when the tag-encoding sequence of tmRNA is engineered. It has long been believed that *trans*-translation occurs around the 3′-end of truncated mRNA lacking a stop codon (non-stop mRNA) in the stalled ribosome since publication of the results of an earlier *in vivo* study using an artificial mRNA (Keiler et al., 1996). Non-stop mRNA can be produced either unexpectedly or in a programmed fashion, and a similar situation can also arise when the normal termination codon is read through in the presence of a non-sense suppressor tRNA (Ueda et al., 2002) or a miscoding drug (Abo et al., 2002). Proteomic analyses have identified endogenous *trans*-translation products from various bacterial sources, indicating that *trans*-translation preferentially occurs at specific sites of specific mRNAs (Roche and Sauer, 2001; Collier et al., 2002; Fujihara et al., 2002; Hong et al., 2007; Barends et al., 2010). Consequently, several situations that promote *trans*-translation in the middle of mRNA have been focused on: translational pausing due to a rare codon (Roche and Sauer, 1999), an inefficient termination codon (Roche and Sauer, 2001; Hayes et al., 2002a; Sunohara et al., 2002) and a programmed stalling sequence (Collier et al., 2004) induces *trans*-translation. However, whether *trans*-translation actually occurs in the middle of mRNA without cleavage has been controversial. It has been found that a bacterial toxin, RelE, cleaves an mRNA specifically at the A-site in the stalled ribosome (Pedersen et al., 2003). RelE is usually inactivated by an antitoxin, RelB, and it is activated by degradation of RelB by Lon protease upon amino acid starvation. Yet, the finding of an A-site-specific endoribonuclease has supported the idea that mRNA cleavage is the prerequisite for *trans*-translation. Ribosome stalling induces cleavage of mRNA at the A-site even in a cell lacking RelE or several other endoribonucleases (Hayes and Sauer, 2003; Sunohara et al., 2004a,b; Li et al., 2008), indicating the involvement of an as-yet-unidentified ribonuclease or the ribosome itself in mRNA cleavage. It has also been reported that the 3′–5′ exoribonulease activity of RNase II is an important prerequisite for A-site-specific mRNA cleavage (Garza-Sánchez et al., 2009). Besides RelE, several kinds of ribosome-dependent endoribonucleases, each having a specific antitoxin, have been identified in *E. coli* (Feng et al., 2013).

*In vitro* studies have clearly shown that *trans*-translation can occur in the middle of mRNA, although the efficiency of *trans*-translation is drastically decreased with increasing length of the 3′-extension from the stalled position (Ivanova et al., 2004; Asano et al., 2005). This is in agreement with results of structural studies showing that the C-terminal tail of SmpB occupies the mRNA path in the early stage of *trans*-translation so that it competes with the 3′-extension of mRNA (Kurita et al., 2010; Neubauer et al., 2012).

Proteomic studies have shown that *trans*-translation preferentially occurs at the proline codon just preceding the stop codon (Hayes et al., 2002a,b). Asp–Pro, Glu–Pro, Pro–Pro, Ile–Pro, and Val–Pro are favorable C-terminal dipeptides for *trans*-translation, suggesting an additional importance of the penultimate residue. In fact, Asp–Pro and Pro–Pro are unusually underrepresented at the C-terminus in most bacterial proteins. Due to the structural irregularity of proline having a secondary amine instead of the primary amine, peptidyl-Pro-tRNA^Pro^ in the A-site would interfere with the access of peptide release factor (Janssen and Hayes, 2009), rather than that of the Ala-tmRNA·SmpB·EF-Tu·GTP complex. Consistently, limited amounts of aminoacyl-tRNA or release factor induce *trans*-translation, indicating competition of *trans*-translation with aminoacyl-tRNA and release factor for sense and non-sense codons, respectively, in the stalled ribosome (Ivanova et al., 2004; Asano et al., 2005; Li et al., 2007). Consecutive proline residues also affect peptidyl-transfer during the elongation process to cause translational arrest, which can be rescued by EF-P (Doerfel et al., 2013; Ude et al., 2013).

These* in vivo* and *in vitro* studies together have settled the controversy shown above: translation can stall even in the middle of mRNA in some situations, and this kind of stalled ribosome can be a potential target for *trans*-translation, although it would substantially occur only after cleavage of mRNA around the A-site by RelE or another as-yet-unidentified ribonuclease with the help of a 3′–5′ exoribonuclease RNase II.

### *Trans*-Translation as Quality Control Systems of Protein and mRNA

As described above, the most significant role of* trans*-translation is to promote recycling of stalled ribosomes in the cell. *Trans*-translation is thought to have additional roles as quality control systems of protein and mRNA.

**FIGURE 4:**
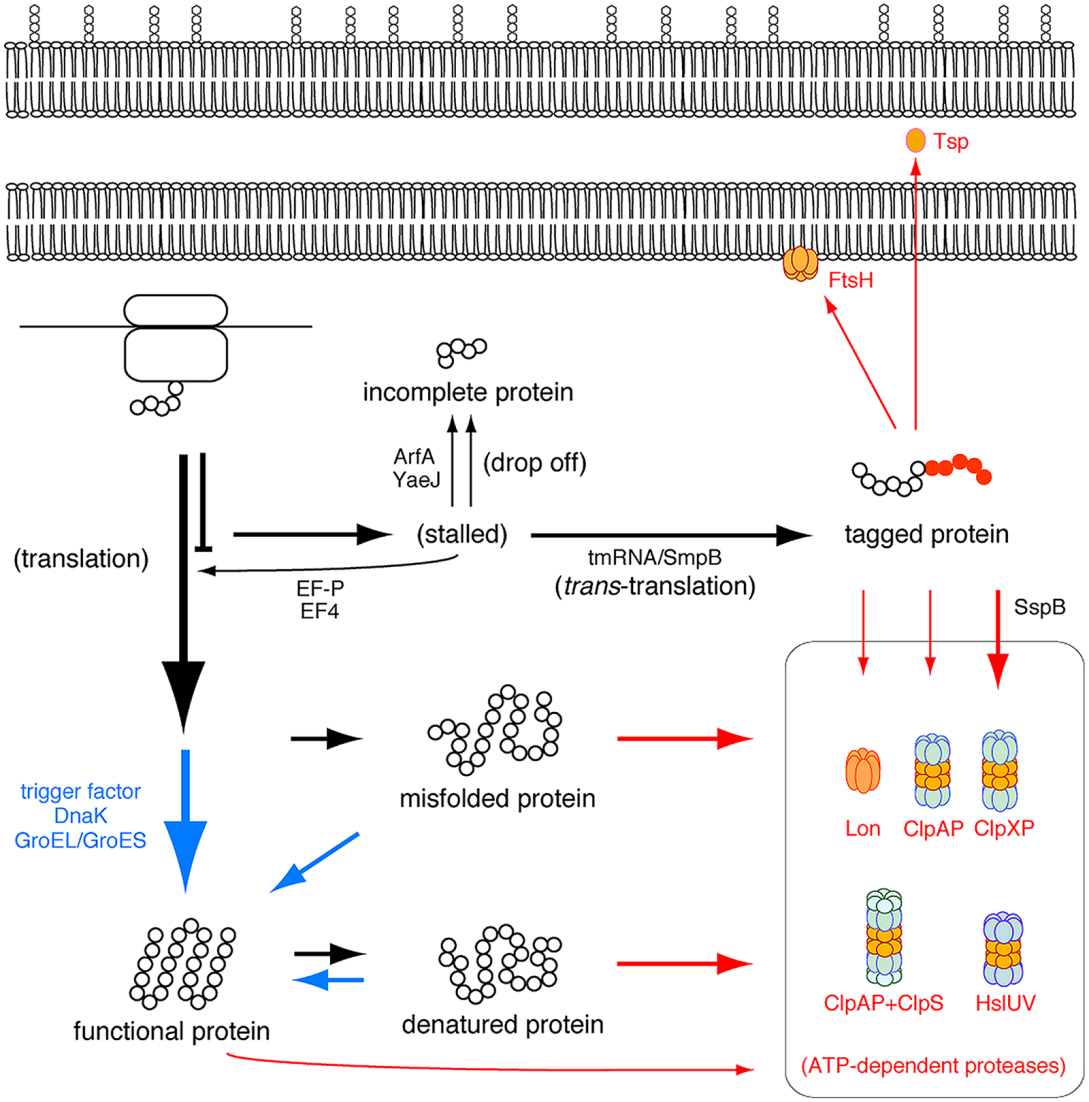
**Ribosome rescue pathways together with folding and degradation pathways in an *E. coli* cell.** Folding and degradation pathways are shown in blue and red, respectively.

Most *trans*-translation products would be non-functional, and thus their accumulation might be deleterious for the cell. To avoid this situation, the tag-peptide and in turn the *trans*-translation products are promptly degraded in the cell by cytoplasmic ATP-dependent proteases (AAA^+^ proteases), including ClpXP, ClpAP, Lon and FtsH, and the periplasmic protease Tsp (Prc; **Figure 4**). ClpX or ClpA recognizes the C-terminal ALAA sequence of the tag-peptide to unfold the *trans*-translation products in an ATP-dependent fashion for degradation by its partner ClpP peptidase (Gottesman et al., 1998). The tag-peptide specifically binds to a protein, SspB, to increase its affinity to ClpX, and consequently ClpXP is thought to play the dominant role in degradation of *trans*-translation products at least in β- and γ-proteobacteria (Flynn et al., 2001) and perhaps in α-proteobacteria (Lessner et al., 2007). Lon participates in degradation of *trans*-translation products under stressful conditions (Choy et al., 2007). FtsH is anchored to the cytoplasmic side of the inner membrane to degrade the membrane-associated *trans*-translation products (Herman et al., 1998). The C-terminal ALAA sequence of the tag-peptide required for ClpXP and ClpAP is highly conserved among bacteria except *Mycoplasma*, in which the tag-peptide terminates with AFA instead of ALAA. This can be addressed by the absence of ClpXP, ClpAP, and Tsp in *Mycoplasma* (Gur and Sauer, 2008; Ge and Karzai, 2009).

While *trans*-translation is induced by cleavage of mRNA in the stalled ribosome as described above, *trans*-translation also promotes further degradation of non-stop mRNA (Yamamoto et al., 2003). *Trans*-translation would expose the 3′ end of non-stop mRNA sequestered by the stalled ribosome, facilitating the access of 3′–5′ exoribonuclease for degradation of non-stop mRNA. It should be a great advantage for the cell, considering that ribosome stalling would be repeated until non-stop mRNA is degraded, even if the stalled ribosome at the 3′ end of the polysome is rescued by *trans*-translation. RNase R is a likely candidate for such an exoribonuclease (Oussenko et al., 2005; Mehta et al., 2006; Richards et al., 2006; Ge et al., 2011). *E. coli* RNase R makes a complex with tmRNA and SmpB (Karzai and Sauer, 2001) via direct interaction with SmpB (Liang and Deutscher, 2010). It is induced under stress conditions in *E. coli* (Chen and Deutscher, 2005) and is involved in cell cycle-regulated degradation of tmRNA in *Caulobacter crescentus* (Hong et al., 2005). *E. coli* RNase R is acetylated in exponential phase, resulting in the exponential phase-specific degradation via tighter binding to tmRNA·SmpB (Liang et al., 2011).

### Physiological Significance of *Trans*-Translation

The apparent universality of the *trans*-translation system among bacteria suggests some biological significance of this system. Indeed, it is essential for some bacteria including *Neisseria gonorrhoeae* (Huang et al., 2000),* Mycoplasma genitalium* (Hutchison et al., 1999), *Haemophilus influenzae* (Akerley et al., 2002), *Helicobacter pylori* (Thibonnier et al., 2008), and *Shigella flexneri* (Ramadoss et al., 2013a), and its depletion causes a wide variety of disorders. Since its lack causes avirulence of some infectious bacteria, the *trans*-translation system has been focused on as an effective target for antibiotics (Shi et al., 2011; Ramadoss et al., 2013b).

Many of these defective phenotypes are caused by a defect in the *trans*-translation reaction rather than degradation of the *trans*-translation products (Keiler, 2008). This suggests that ribosome recycling is more important for the cell than preventing accumulation of non-functional proteins. Upon starvation of amino acids, supply of amino acids from *trans*-translation products should become important for new protein synthesis (Pedersen et al., 2003; Li et al., 2008).

*Trans*-translation is often employed for regulation of gene expression. In *E. coli*, tmRNA-mediated *trans*-translation targets mRNA for LacI, a repressor of the lactose operon, to accelerate its degradation upon glucose depletion, leading to derepression of the *lac* operon (Abo et al., 2000). In *Bacillus subtilis*,* trans*-translation occurs around the catabolite responsive element (*cre*) sequence, a binding site of the repressor protein catabolite control protein A (CcpA), within the coding region of several mRNAs including TreP mRNA for trehalose phosphorylase (Fujihara et al., 2002). Binding of CcpA to the *cre* sequence would induce a transcriptional roadblock to produce truncated mRNA (Ujiie et al., 2009). In *C. crescentus*, the cell cycle (Keiler and Shapiro, 2003a) and the initiation of DNA replication (Keiler and Shapiro, 2003b; Hong et al., 2007) are controlled by *trans*-translation.

There is accumulating evidence for increased importance of tmRNA under stressful conditions, such as high or low temperature (Oh and Apirion, 1991; Muto et al., 2000; Shin and Price, 2007), nutrient starvation (Oh and Apirion, 1991; Okan et al., 2006; Abe et al., 2008), ethanol treatment (Muto et al., 2000), cadmium treatment (Muto et al., 2000), and acid exposure (Thibonnier et al., 2008). Stresses might increase the frequency of aberrant translation in cells, which can be rescued by *trans*-translation. Consistently, the total amount of *trans*-translation products increases under stressful conditions (Fujihara et al., 2002). Perhaps in response to the increased requirement of the *trans*-translation system, the intracellular level of tmRNA or SmpB increases with an increase in stress in some bacteria (Muto et al., 2000; Palecková et al., 2007; Rezzonico et al., 2007).

The *trans*-translation system sometimes regulates other stress response systems possibly via expression of a global regulator. For example, depletion of tmRNA induces heat shock response in *E. coli* (Munavar et al., 2005). The expression level of the sigma factor RpoS (sigma S), which controls the expression of a series of genes involved in general stress response, is positively controlled by *trans*-translation in *E. coli* (Ranquet and Gottesman, 2007). The involvement of *trans*-translation in the extracellular stress-response pathway via another sigma factor, RpoE (sigma E), has also been suggested (Ono et al., 2009). Other stress-related proteins including the molecular chaperone DnaK are regulated by *trans*-translation in streptomycetes (Barends et al., 2010). Interestingly, the expression of ArfA, an alternative ribosome rescue system (described in a later subsection), is regulated by *trans*-translation (Chadani et al., 2011a; Garza-Sánchez et al., 2011).

### Evolutionary Aspects of *Trans*-Translation

Although it is not essential for viability in most bacteria, tmRNA is present universally in the bacterial kingdom and in some plastids or mitochondria of some protists. The tRNA-like secondary structure of TLD as well as several tRNA-specific consensus sequences is highly conserved except for the deformed D-arm structure, which has an extensive interaction with SmpB. In addition, the third base-pair position of the amino acid acceptor stem is completely conserved as G–U, which serves as a potent identity determinant for recognition by alanyl-tRNA synthetase (AlaRS). Alanine might not be an absolute prerequisite for *trans*-translation as the amino acid aminoacylated to tmRNA as exemplified by Nameki et al. (1999a). However, AlaRS might be the most preferable aminoacyl-tRNA synthetase for tmRNA, considering the unique recognition mode of AlaRS depending largely on the acceptor stem instead of the anticodon. The tRNA-like structure would also be significant for recognition by EF-Tu and RNase P as well as for ribosome binding. In contrast to the high degree of conservation of TLD, there is variation in the pseudoknot-rich region (Nameki et al., 1999b; Williams, 2002). Plastid tmRNA has fewer or no pseudoknot structures (Gueneau de Novoa and Williams, 2004). Indeed, at least the last three of four pseudoknots of *E. coli* tmRNA are dispensable for *trans*-translation *in vitro* (Nameki et al., 2000), although they participate in proper folding and processing of tmRNA (Wower et al., 2004). TLD and the pseudoknot-rich region are linked by a long degenerated helix, which protrudes from TLD in a direction corresponding to that of the long variable arm of class II tRNA (**Figure 1B**; Bessho et al., 2007). This direction should be conserved within the constraints of the tRNA-like dynamic behavior of tmRNA in the limited space of the ribosome, although the sequence of the connector helix is less conserved.

In some lineages of α-proteobacteria, β-proteobacteria, cyanobacteria, and mitochondria of lower eukaryotes, tmRNA is separated into two pieces, a 5′-coding piece typically including only two pseudoknots (PK1 and PK2) with the tag-encoding region in between and a 3′-amino acid acceptor piece, and the two pieces join together by base-pairing to form into a tRNA-like structure similar to that in one-piece tmRNA (**Figure 1A**; Keiler et al., 2000; Williams, 2002; Sharkady and Williams, 2004). The gene for two-piece tmRNA is circularly permuted (Keiler et al., 2000; Williams, 2002; Mao et al., 2009), and the permuted precursor might be processed into a mature two-piece tmRNA probably with the help of RNase P and tRNase Z. A similar processing strategy has been found in the circularly permuted tRNA gene in some primitive eukaryotes or archaebacteria (Soma et al., 2007). The two-piece tmRNA in *C. crescentus* belonging to α-proteobacteria has been shown to actually function in* trans*-translation (Keiler et al., 2000). Either one-piece or two-piece tmRNA is present in mitochondrial genome of some groups of protists (jakobids; Jacob et al., 2004). They lack a tag-encoding sequence as well as pseudoknots, arguing against their capacity for bacterial type of *trans*-translation.

SmpB together with tmRNA is universally present in bacteria. Plastid tmRNA is encoded by the plastid genome, while plastid SmpB is encoded by the nuclear genome and it is imported from the cytoplasm (Jacob et al., 2005). Up to now, there has been no report about mitochondrial SmpB. Both tmRNA and SmpB should have been required at the birth of *trans*-translation. The gene for tmRNA might have been formed by insertion of something into a tRNA^Ala^ gene. In contrast, no one can envisage the origin of SmpB because of the absence of its homologue.

### Diversity of Rescue Systems of Stalled Translation

As described above, *trans*-translation targets various kinds of translational pausing due to a rare codon, an inefficient termination codon or a programmed stalling sequence, but after cleavage of mRNA. The bacterial cell has alternative mechanisms to rescue the stalled ribosome (**Figure 4**; Himeno, 2010).

Peptidyl-tRNA hydrolase (Pth) has an activity to hydrolyze the linkage between tRNA and the nascent polypeptide of peptidyl-tRNA after it drops off from the ribosome. Drop-off is enhanced by RRF alone, RRF together with RF3 (Heurgué-Hamard et al., 1998; Gong et al., 2007) or RRF, IF3, and EF-G (Singh et al., 2008). Drop-off was initially assumed to occur in the earlier cycles of elongation. This seems reasonable considering that a longer nascent polypeptide chain would prevent release of peptidyl-tRNA from the peptide channel of the translating ribosome. However, drop-off has been shown to efficiently occur at the 3′ end of non-stop mRNA in the absence of tmRNA (Kuroha et al., 2009). Overexpression of tmRNA suppresses the temperature-sensitive phenotype of Pth (Singh and Varshney, 2004), suggesting that Pth contributes not only to hydrolyzing the dropped-off peptidyl-tRNA but also to rescuing the stalled ribosome or suggesting that *trans*-translation can substitute for spontaneous or factor-promoting drop-off and the following peptidyl-tRNA hydrolysis by Pth.

It has recently been found that two proteins, ArfA (YhdL) and YaeJ (ArfB), facilitate rescue of the stalled ribosome. A single knockout of either *E. coli* ArfA or tmRNA is viable, whereas a double knockout is lethal, explaining why tmRNA is not essential in many bacteria (Chadani et al., 2010). The ribosome rescue activity of ArfA was initially shown using *E. coli *crude extract (Chadani et al., 2010). However, ArfA alone does not have an activity to hydrolyze peptidyl-tRNA in the P-site possibly due to the absence of a typical GGQ catalytic motif, and it requires the help of RF-2 (Chadani et al., 2012; Shimizu, 2012). RF-2 usually acts as a UAA or UGA codon-dependent release factor, while it serves as a stop codon-independent release factor in the presence of ArfA. Intriguingly, translation for ArfA protein is stalled near the 3′-terminus of its mRNA due to cleavage by RNase III, and consequently ArfA is usually degraded via the *trans*-translation system, and only when the cellular *trans*-translation activity becomes diminished, is C-terminally truncated but active ArfA synthesized via spontaneous drop-off or ArfA-mediated release of the nascent polypeptide (Garza-Sánchez et al., 2011; Chadani et al., 2011a). Thus, the ArfA-mediated ribosome rescue system is considered to be a backup system for *trans*-translation. YaeJ has also been shown to rescue the ribosomes stalled at the 3′ end of a non-stop mRNA *in vitro* (Handa et al., 2011) and *in vivo* (Chadani et al., 2011b). A double knockout of *E. coli* ArfA and tmRNA is lethal as described above, whereas overexpression of YaeJ makes it viable (Chadani et al., 2011a). Unlike ArfA, YaeJ alone has an activity to hydrolyze peptidyl-tRNA in the P-site of the stalled ribosome, as expected from the similar sequence and structure to those of the catalytic domain of bacterial class I release factor having a GGQ motif. YaeJ is likely to act as a stop codon-independent peptide chain release factor since it lacks a stop codon-recognition domain and instead it is replaced by a C-terminal basic-residue-rich extension that might be unstructured in solution (Handa et al., 2010). In a crystal structure of *E. coli *YaeJ in complex with the stalled ribosome from *T. thermophilus*, the C-terminal extension of YaeJ, like that of SmpB, binds along the mRNA path of the stalled ribosome extending to the downstream mRNA tunnel with an α-helical structure (Gagnon et al., 2012). ArfA as well as Ala-tmRNA·SmpB·EF-Tu(GTP) does not favor the long 3′ extension of mRNA from the decoding region upon entrance to the stalled ribosome, while YaeJ is less sensitive (Shimizu, 2012). Thus bacterial cells are equipped with multiple systems to cope with stalled translation, and they are therefore often still viable even when they lose the *trans*-translation system. Judging from phenotypes of factor-depleted cells, the *trans*-translation system must be the primary ribosome rescue system.

There are some reports about stress-specific ribosome rescue systems. The heat shock protein HSP15 has been shown to bind to the dissociated 50S subunit with a nascent protein (Korber et al., 2000). Upon exposure to a high temperature, a fraction of translating ribosomes might prematurely be dissociated into subunits, although peptidyl-tRNA remains bound to the dissociated 50S subunit unless the nascent peptide is short. In this 50S subunit, HSP15 fixes peptidyl-tRNA at the P-site to make the A-site free presumably for entrance of a peptide release factor (Jiang et al., 2009). High intracellular magnesium ion concentration or low temperature causes translational arrest after defective translocation. It also promotes release of a GTPase, EF4 (LepA), which is usually stored in the cell membrane, into the cytoplasm to rescue the translational arrest by back translocation (Pech et al., 2011).

Translation often stalls at a specific site on an mRNA in a programmed fashion. As in the case of ArfA expression described above (Chadani et al., 2011a; Garza-Sánchez et al., 2011), translational arrest is sometimes used for repression of gene expression via *trans*-translation. On the other hand, a stalled ribosome often prevents access of rescue machineries to keep translational arrest for regulation of gene expression. *E. coli* tryptophanase (*tna*) operon is induced by tryptophan via the translational arrest of the leader peptide (TnaC) by inhibiting hydrolysis of peptidyl-tRNA^Pro^ by RF2 (Yanofsky, 2007). This stalled ribosome is not rescued by *trans*-translation in the presence of tryptophan, although it is rescued slowly by RRF and RF3, leading to drop-off (Gong et al., 2007). The ribosome is also stalled at an internal proline codon of *E. coli* *secM* mRNA, which up-regulates the translation of the downstream SecA-encoding sequence presumably by disrupting the secondary structure that sequesters the ribosome binding site (Muto et al., 2006). This translational arrest is caused by inefficient peptidyl-transfer of Pro-tRNA^Pro^ in the A-site to the nascent peptidyl-tRNA in the P-site, which inhibits entrance of Ala-tmRNA to the A-site and the A-site specific cleavage of mRNA (Garza-Sánchez et al., 2006). The translation of *B. subtilis* *yidC* mRNA is regulated by translational arrest at multiple sites on the upstream *mifM* mRNA (Chiba et al., 2009; Chiba and Ito, 2012). Puromycin is less reactive to this translational arrest, suggesting that ribosome rescue machineries including Ala-tmRNA·SmpB·EF-Tu(GTP) are also less accessible to the A-site of this stalled ribosome. Consecutive proline codons cause a translational arrest due to inefficient peptidyl-transfer between peptidyl-(Pro)n-tRNA in the P-site and Pro-tRNA in the A-site (Doerfel et al., 2013; Ude et al., 2013). In this case, the A-site is occupied by Pro-tRNA^Pro^, and in turn Ala-tmRNA·SmpB·EF-Tu(GTP), ArfA or YaeJ would fail to access this stalled ribosome. Instead, the peptidyltransferase center is modulated by EF-P binding to the region between the P-site and E-site to resume peptidyl-transfer (Blaha et al., 2009). Pro–Pro–Pro and Gly–Pro–Pro arrest sequences, which can be rescued by EF-P, are often found in bacterial genes, and they might be programmed for regulation of gene expression.

Pth is essential for bacteria and is widely distributed among the other domains. While the *trans*-translation system universally exists in bacteria, YaeJ is distributed among Gram-negative bacteria and ArfA shows more limited distribution within enterobacteria. EF-P is conserved among bacteria and its homologue (a/eIF5A) is universally present in archaea and eukaryotes. EF4 is universally conserved among bacteria. Neither tmRNA nor SmpB is present in the cytoplasm of eukaryotes, where a complex of Dom34p (Pelota) and the GTP-binding protein Hbs1 promotes subunit dissociation of the stalled ribosome and drop-off of peptidyl-tRNA (Shoemaker et al., 2010) in concert with an ATPase, ABCE1 (Pisareva et al., 2011). The Dom34p·Hbs1 complex is structurally similar to the eRF1·eRF3 complex or the aminoacyl-tRNA·EF-Tu complex (Chen et al., 2010), although the GGQ motif is absent in Dom34, and peptidyl-tRNA hydrolysis is assumed to be catalyzed by Pth after drop-off. In mitochondria, two protein factors partially similar to the bacterial class I release factor, ICT1 (a homologue of YaeJ) and C12orf65, both lacking a stop codon-recognition domain while retaining the catalytic GGQ motif, participate in ribosome rescue (Handa et al., 2010; Richter et al., 2010; Kogure et al., 2012, 2014). ICT1 (YaeJ), but not C12orf65, has an insertion of an α-helix in the GGQ domain, and thus ICT1 is less similar to class I release factor. C12orf65 has been found in very limited bacteria. Collectively, various kinds of release factor homologues, YaeJ, ICT1, C12orf65, ArfA/RF2, Dom34p (Pelota), and Hbs1, have been found to participate in ribosome rescue. The *trans*-translation system is unique in that it employs a small RNA and that it prevents accumulation of non-functional proteins from truncated mRNA in the cell.

## Conflict of Interest Statement

The authors declare that the research was conducted in the absence of any commercial or financial relationships that could be construed as a potential conflict of interest.
